# Potent bicyclic inhibitors of malarial cGMP-dependent protein kinase: approaches to combining improvements in cell potency, selectivity and structural novelty

**DOI:** 10.1016/j.bmcl.2019.08.014

**Published:** 2019-10-01

**Authors:** Jonathan M. Large, Kristian Birchall, Nathalie S. Bouloc, Andy T. Merritt, Ela Smiljanic-Hurley, Denise J. Tsagris, Mary C. Wheldon, Keith H. Ansell, Peter J. Coombs, Catherine A. Kettleborough, David Whalley, Lindsay B. Stewart, Paul W. Bowyer, David A. Baker, Simon A. Osborne

**Affiliations:** a Centre for Therapeutics Discovery, LifeArc, Accelerator Building, Open Innovation Campus, Stevenage SG1 2FX, UK; b Faculty of Infectious and Tropical Diseases, London School of Hygiene & Tropical Medicine, Keppel Street, London WC1E 7HT, UK

**Keywords:** Malaria, *Plasmodium falciparum*, cGMP, Protein kinase G, Imidazopyridine, SAR

## Abstract

Focussed studies on imidazopyridine inhibitors of *Plasmodium falciparum* cyclic GMP-dependent protein kinase (*Pf*PKG) have significantly advanced the series towards desirable *in vitro* property space. LLE-based approaches towards combining improvements in cell potency, key physicochemical parameters and structural novelty are described, and a structure-based design hypothesis relating to substituent regiochemistry has directed efforts towards key examples with well-balanced potency, ADME and kinase selectivity profiles.

## Graphical abstract

**Figure:**
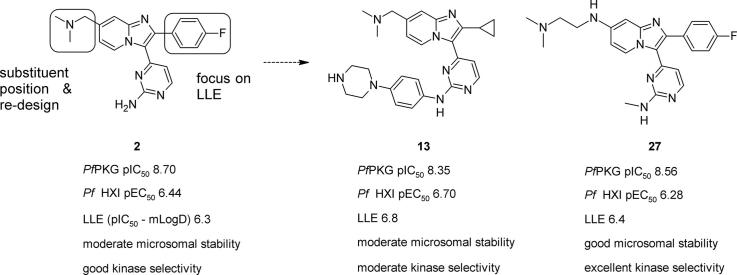

Malaria is one of the most prevalent infectious diseases of the developing world in humans, whose causative agent is the protozoan parasite *Plasmodium*, with most deaths caused by *P. falciparum*. Despite being largely preventable and treatable, it was responsible for 435,000 deaths in 2017; young children and pregnant women in sub-Saharan Africa are particularly at risk.^1^ In addition to continuing challenges in the contexts of policy development and socio-economic impact,^2^ the observation of increasing resistance to current standard-of-care treatments is significant. This is driving research and development efforts to uncover new mechanisms by which the disease can be controlled and prevented.^3^

Studies on the malarial kinome continue to provide well characterised and credible new targets for antimalarial small molecule drug discovery.^4^, ^5^ The cGMP-dependent kinase *Pf*PKG is one kinase which meets many of the criteria for such a target. Pharmacological characterisation using early chemical inhibitors in combination with reverse genetics has demonstrated the important role of this enzyme in numerous critical processes in the malaria life cycle.^6^, ^7^, ^8^, ^9^, ^10^, ^11^ Following previous experience with progressing chemical inhibitors of other important malarial kinases,^12^, ^13^, ^14^, ^15^ we have recently begun to disclose our efforts to develop a series of *Pf*PKG inhibitors based upon both bicyclic^16^ and monocyclic scaffolds.^17^ In the bicyclic series, a number of advanced analogues were shown to possess promising *in vitro* activity, a well-defined mechanism of action and property profiles which translated to target-driven efficacy *in vivo*.^16^ An ongoing objective is to develop this chemical series with a view to improving key physiochemical parameters and compound novelty whilst retaining cell potency and lipophilic ligand efficiency (LLE).^19^

A recent report from us described initial efforts towards these goals by evaluating the aminopyrimidine hinge binding motif, bicyclic core structure and basic substituent positioning.^20^ Investigation of each of those structural features was found to be both necessary and productive, and the resulting compound profiles pointed strongly to retaining these motifs in their original forms. As a result, the profiles of analogues such as **1** (Figure 1) challenged us to consider additional strategies for re-positioning the series in suitable ADME property space whilst maintaining suitable levels of *in vitro* activity and improving compound novelty. A first approach was to reduce the size of the 4-fluorophenyl motif to lower lipophilicity and hence increase lipophilic ligand efficiency (LLE)^19^ (Figure 1 – A). Previous SAR^20^ suggested potency could be regained by enlarging the pyrimidine substituent, if needed (Figure 1 – B). In a second set of analogues, it was anticipated that re-design of the benzylic dimethylaminomethyl side chain in the prototypical inhibitor compound **2** would enable lowering of logD (for example by increasing chain length and basicity) and could also address one likely point of metabolic liability (for example by replacing the benzylic carbon atom with a heteroatom) (Figure 1 – C). Here we discuss the results of these investigations and show their significant beneficial impact against the above criteria.

**Figure 1:**
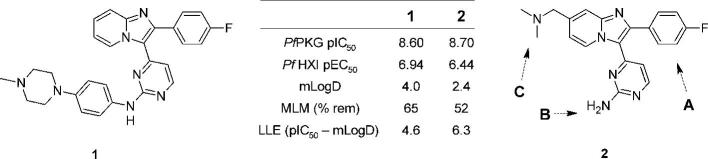
*In vitro* profiles of imidazopyridine **1** and **2**, and design modifications to be applied to **2**: A – truncate the aryl group; B – enlarge the pyrimidine substituent if required; C – re-design the basic substituent. ADME data: mLogD = measured logD; MLM = % remaining after 30 min incubation with mouse liver microsomes.

We first examined the possibility of improving the lipophilic efficiency by focusing on the large 4-fluorophenyl motif, and initially retained the original basic substituent at the 7-position of the bicyclic core in doing so. The main design emphasis was to attempt to balance the size of the pyrimidine substituent with a smaller lipophilically efficient replacement for the 4-fluorophenyl group. Among a small set of initial replacements, prepared by the general route shown in Scheme 1, the cyclopropyl analogue **9** was of lower potency in a biochemical assay^21^ as compared to **2**, but significantly also showed a lower mLogD value of 1.7 of 1.7 (Table 1).^22^ Given that potency was lower than desirable, further analogues incorporating the cyclopropyl group were designed to combine a lower mlogD with improvements in potency and LLE. Hence a set of compounds with larger groups appended to the aminopyrimidine nitrogen was prepared using variations of the same chemical approach. Small alkyl groups such as that in **10** did not provide any further boost in activity or LLE but, in line with previous SAR, arylaminopyrimidines such as **11** and **12** were more biochemically active and possessed the anticipated trend towards lower mLogD. The most balanced profile was achieved in **13**,^23^ which showed similar levels of both biochemical potency and anti-malarial activity in a blood stage hypoxanthine incorporation (HXI) cell assay^21^ compared to **2**, coupled with improvements in mLogD and LLE.

**Scheme 1:**
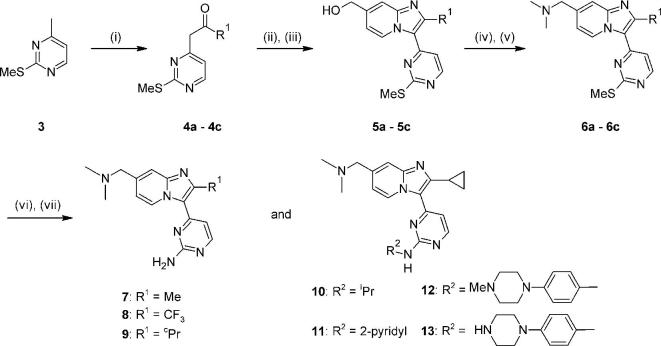
*Reagents and conditions*: (i) LiHMDS, R^1^CO_2_Et, THF, −78 °C – rt, 3 h, 27–76%; (ii) Bu_4_NBr_3_ or NBS, CH_2_Cl_2_, rt, 2 h; (iii) 2-aminopyridine-4-methanol, EtOH, 4 Å sieves, 100 °C, 18 h, 12–44% for two steps; (iv) MsCl, Et_3_N, THF, 0 °C, 1 h or SOCl_2_, CH_2_Cl_2_, 50 °C, 1 h; (v) Me_2_NH, THF, 0 °C – rt, 33–65% for two steps; (vi) H_2_O_2_, Na_2_WO_4_·2H_2_O, AcOH, MeOH, 0 °C – rt, 3 h; (vii) for **7**–**9**: NH_4_OAc, melt, 130 °C, 3 h, 5–27% for two steps; for **10**: ^i^PrNH_2_ (neat), 60 °C 3 h, 23% for two steps; for **11**: 2-aminopyridine (excess), NMP, microwave, 150 °C, 3 h, 8% for two steps; for **12**: 4-(4-methylpiperazino)aniline, neat, microwave, 170 °C, 15 min, 5% for two steps; for **13**: 4-(4-aminophenyl)piperazine-1-carboxylic acid *tert*-butyl ester, TFA, ^s^BuOH, 110 °C, 6 h, then TFA, CH_2_Cl_2_, rt, 2 h, 10% for three steps.

**Table 1:** Replacing the 4-fluorophenyl group with a cyclopropyl motif.

**Figure:**
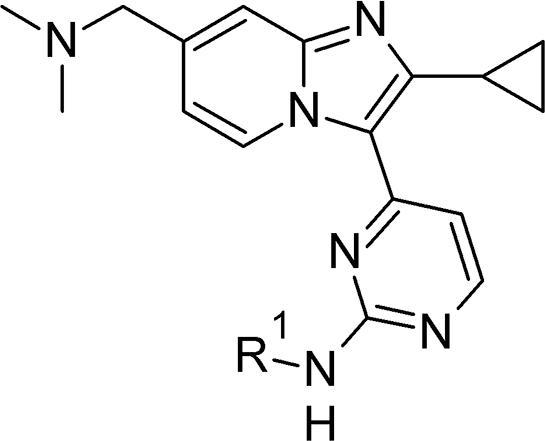

Compound	R^1^	*Pf*PKG pIC_50_	*Pf* HXI pEC_50_^a^	LLE	mLogD
**2**	–	8.70	6.44	6.3	2.4
**9**	H	7.45	*nt*	5.8	1.7
**10**		7.36	*nt*	4.4	3.0
**11**		8.06	5.76	5.8	2.5
**12**		8.14	6.95	6.0	2.1
**13**		8.35	6.70	6.8	1.5

a *nt* = not tested.

Turning next to the basic substituent on the bicyclic core, a small number of molecules were initially designed to identify the optimum position at which to locate this motif. We decided to employ the benzylic dimethylaminomethyl group present in **2** for this analysis. Docking of **2** and its 5-, 6- and 8- regioisomers into an apo-structure of *Pf*PKG (PDB:5DYK^24^) suggested that the best site for that substituent was at the 7-position (Figure 2). The location of the positively charged basic center between two acidic protein residues (E625 and D682) was judged to be optimal for that particular group. Whilst relocating to the 6- or 8- positions appeared to be spatially tolerable, sub-optimal interaction with the acids and a subsequent loss in affinity was predicted. Appending several possible groups at the 5-position appeared to result in a significant steric clash with the pyrimidine hinge binding motif (data not shown); this was predicted to cause a significant loss of activity and hence was not pursued.

**Figure 2:**
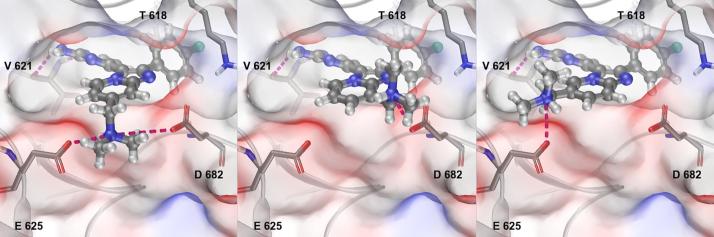
Docking of **2** (left), the 8-regioisomer **15** (centre) and the 6-regioisomer **16** (right) into an apo-*Pf*PKG crystal structure (PDB:5DYK^24^), with protein surface coloured by electrostatic potential. H-bonds and charge interactions are shown as dashed lines.

This hypothesis was tested by synthesizing the 8- and 6-regioisomers **15** and **16** respectively. Using variations of previously described chemical approaches,^18^, ^20^ compounds **15** and **16** could be prepared from the bromoketone building block **14**^25^ (Scheme 2), in good yields over 5 synthetic steps.

**Scheme 2:**
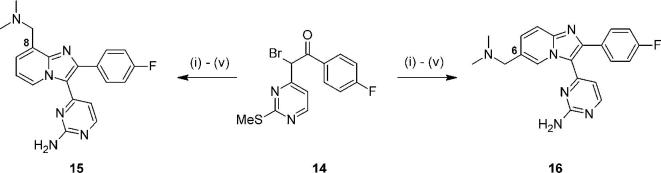
*Reagents and conditions*: (i) 2-aminopyridine-3-methanol (for **15**) or 2-aminopyridine-5-methanol (for **16**), MeCN, NaHCO_3_, 90 °C, 18 h: 32–75%; (ii) MsCl, Et_3_N, THF, 0 °C, 4 h; (iii) Me_2_NH, THF, 0 °C – rt, 3 h, 60–89% for two steps; (iv) H_2_O_2_, Na_2_WO_4_·2H_2_O, AcOH, MeOH, rt, 3 h; (v) NH_4_OAc, melt, 120 °C, 3 h, 19–27% for two steps.

These two compounds showed reductions in their biochemical activity, as compared to **2** (Table 2), which were in line with predictions from the docking studies. Lipophilic ligand efficiency for the 8-substituted compound **15** was also higher than for 6-analogue **16**, in part due to an interesting divergence in mLogD (values of 1.6 for **15**, 2.3 for **16**, as compared to 2.4 for **2**). However, the key factor of lower synthetic accessibility for 8-position analogues emerged, which directed our efforts away from preparing further compounds of this kind. In contrast, the position of the two key acidic residues at the binding pocket mouth implied that re-design of the basic substituent into longer chain variants and appropriate conformationally constrained versions might be productive. This design hypothesis suggested that substituents of these new types at either the 6- or 7-positions should be evaluated.

**Table 2:** Position of basic substituent attachment.

**Figure:**
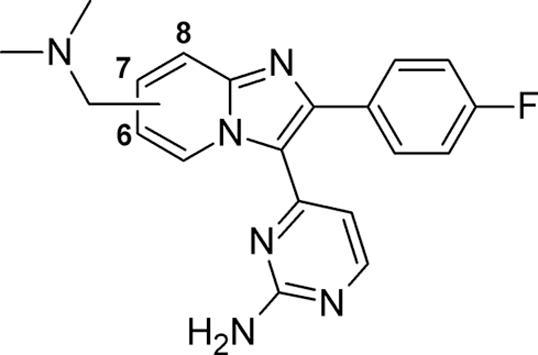

Compound	Substituent position	*Pf*PKG pIC_50_	LLE
**2**	7	8.70	6.3
**15**	8	7.53	5.9
**16**	6	7.29	5.0

We tested this proposal by making compounds bearing such modifications to the dimethylaminomethyl side chain in **2**, and chose to include adjustments in both expected pKa and conformation by varying the linking atom, chain length and ring size in new analogues. Preparation of key intermediates **17**–**19** in three steps, followed by palladium-catalysed aminations or microwave-mediated direct displacements provided the *N*-linked and O-linked examples **20–27** respectively (Scheme 3).^26^ The 7-C-linked analogue **29** was also prepared in four synthetic steps from intermediate **28**, which was itself constructed by condensing bromoketone **14** with the appropriate building block 2-(2-aminopyridin-4-yl)ethan-1-ol.

**Scheme 3:**
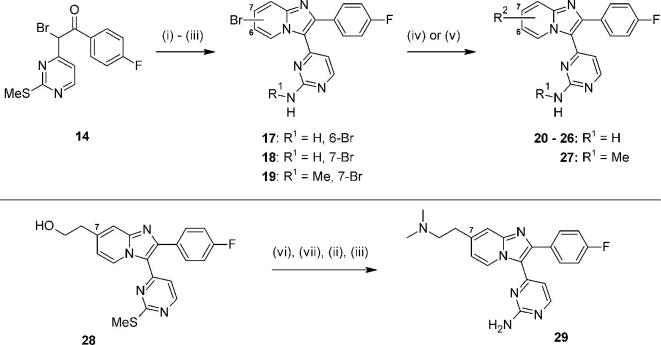
*Reagents and conditions*: (i) 2-amino-4-bromopyridine or 2-amino-5-bromopyridine, EtOH, 4 Å sieves, 80 °C, 18 h, 20–36%; (ii) H_2_O_2_, Na_2_WO_4_·2H_2_O, AcOH, MeOH, rt – 50 °C, 18 h; (iii) For R^1^ = H: NH_4_OAc, melt, 130 °C, 18 h, 30–52% for two steps; for R^1^ = Me: Me_2_NH, THF, 70 °C, 18 h, 56% for two steps; (iv) for R^2^ = amine: Pd(OAc)_2_, JohnPhos, R_2_NH, NaO*^t^*Bu, dioxane, 100 °C, 18 h, 3–29%; (v) for R^2^ = alcohol: KO*^t^*Bu, ROH, NMP, microwave, 170 °C, 10 min, 6%; (vii) MsCl, Et_3_N, THF, 0 °C, 1 h; (vi) Me_2_NH, THF, 60 °C, 10 h, 49% for two steps.

These analogues (along with the earlier example **16**) demonstrated that extending *via* a conformationally constrained basic group (as in **20**) or *via* an open chain version (as in **21**) at either the 7- or 6-position of the core could each provide good biochemical potency (Table 3). Neither of these compounds appeared to possess a particular advantage in any aspect of their *in vitro* profiles as compared to **2**. Interestingly, the related pair of piperazine regioisomers **22** and **23** showed a subtle contrast in mLogD, where the 6-isomer **23** was found to possess the lower value. The cell activity of **23** was also slightly lower as compared to **22**. Synthetic access to 6-substituted compounds was also found to be generally less efficient; considering this and other contributing factors,^27^ we decided to focus additional efforts on 7-linked analogues only.

**Table 3:** Basic side chain variations.

**Figure:**
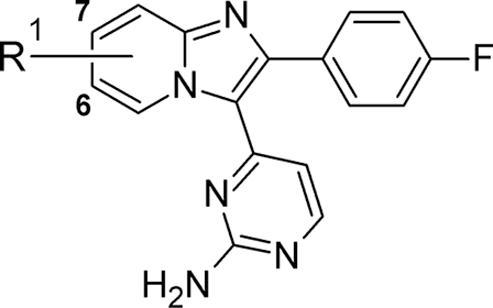

Compound	R^1^	*Pf*PKG pIC_50_	*Pf* HXI pEC_50_^a^	LLE	mLogD
**2**	7-CH_2_NMe_2_	8.70	6.44	6.3	2.4
**16**	6-CH_2_NMe_2_	7.29	*nt*	5.0	2.3
**20**	7-	8.07	6.07	6.0	2.1
**21**	6-NH(CH_2_)_3_NMe_2_	7.90	*nt*	5.7	2.2
**22**	7-	8.48	6.58	5.4	3.1
**23**	6-	8.58	6.06	5.8	2.8

a *nt* = not tested.

Using the same synthetic chemistry as shown in Scheme 3, a small additional set of 7-substituted analogues was prepared and evaluated (Table 4). As compared to **20**, increasing the ring size and hence altering the conformational constraint in **24** gave modest improvements in biochemical potency and cell activity, though only a slight change to LLE. Microsomal stability was improved significantly, perhaps due to constraining the conformation in the basic side chain. For the open chain examples **25** and **29**, *in vitro* ADME profiles very similar to **2** could be obtained, though both showed lower biochemical activity (and hence no further benefit in LLE) and microsomal stability had not improved. Both LLE and microsomal stability could be improved by returning to a nitrogen-linked design in the open chain analogue **26**, for which the essentially unchanged mLogD (as compared to **25** and **29**) was accompanied by better biochemical potency and LLE. Finally, positioning an additional carbon atom within the aminopyrimidine group gave **27**; this notable compound showed an excellent balance of good biochemical potency, *in vitro* activity against the parasite and improved LLE and mLogD values. The effect of the secondary aminopyrimidine (in **27**) on microsomal stability, relative to the primary aminopyrimidine (in **26**), was also significant.

**Table 4:** Basic substituents at the 7-position.

**Figure:**
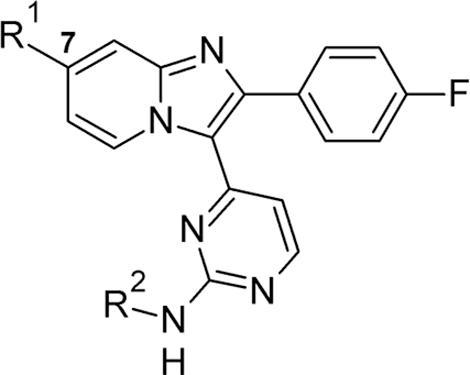

Compound	R^1^	R^2^	*Pf*PKG pIC_50_	*Pf* HXI pEC_50_^a^	LLE	mLogD	MLM % rem^b^
**2**	7-CH_2_NMe_2_	H	8.70	6.44	6.3	2.4	52
**24**		H	8.44	6.46	6.2	2.2	88
**25**	7-O(CH_2_)_2_NMe_2_	H	8.13	6.09	5.9	2.2	54
**26**	7-NH(CH_2_)_2_NMe_2_	H	8.59	*nt*	6.5	2.1	80
**27**	7-NH(CH_2_)_2_NMe_2_	Me	8.56	6.28	6.4	2.2	93
**29**	7-(CH_2_)_2_NMe_2_	H	7.69	6.00	5.8	1.9	65

a *nt* = not tested.

b % remaining after 30 min incubation with mouse liver microsomes.

The two most promising compounds identified − **13** and **27** – were profiled and compared *in vitro* (Table 5). In addition to previously described improvements in mLogD and LLE, high kinetic solubility (measured using PBS at pH7.4 as buffer) was maintained in each case and both compounds were shown to be non-cytotoxic. Despite a significantly lower mLogD value, the mouse microsomal stability of **13** surprisingly remained at the same level as for **2**, for which we have no clear explanation.^28^ In particular, **27** matched excellent biochemical and cell potency with significantly higher stability in mouse microsomes to give a highly promising and well-balanced overall profile. Selectivity was assessed by screening **13** and **27** against a human kinase panel^29^ at a single 1 µM concentration (Figure 3). As expected, the smaller cyclopropyl motif in **13** resulted in a decreased level of selectivity, whilst **27** showed an excellent selectivity profile against the kinases screened. We also tested compounds **2** and **27** against the two human orthologues of PKG; no activity was observed up to a top assay concentration of 1 µM,^30^ indicating a high level of selectivity for the malarial kinase.

**Table 5:** Full *in vitro* profiles for compounds **2**, **13** and **27**; ^a^ % remaining after 30 min incubation with mouse liver microsomes; ^b^ kinetic solubility; ^c^*in vitro* cytotoxicity assay measured in HepG2 human liver-derived cells – concentration at which half of cells remained viable at 48 h.^16^

Compound	*Pf*PKG pIC_50_	*Pf* HXI pEC_50_	LLE	mLogD	MLM % rem^a^	Kin sol (µM)^b^	HepG2 pEC_50_^c^
**2**	8.70	6.44	6.3	2.4	52	200	< 4.7
**13**	8.35	6.70	6.8	1.5	53	189	< 5
**27**	8.56	6.28	6.4	2.2	93	207	< 4.7

**Figure 3:**
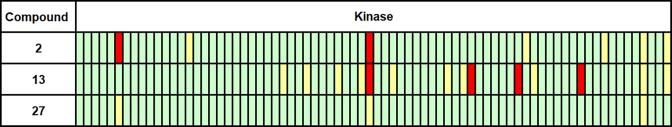
Kinase selectivity data for representative imidazopyridines **2**, **13** and **27** on screening against a human kinase panel at 1 µM concentration; green < 50% inhibition; yellow 50–90% inhibition; red > 90% inhibition.^29^

We have reported here the results of our continuing effort to progress a series of imidazopyridines as inhibitors of *Pf*PKG, focusing on alteration of the 4-fluorophenyl group and re-design of the basic substituent as key strategic aims. By concentrating on cell potency, lipophilic ligand efficiency and structural novelty in tandem, compounds such as **27** in particular were developed to populate a highly desirable and novel area of chemical space as potent, lower molecular weight, lipophilically efficient analogues with improved *in vitro* ADME and selectivity profiles. Studies towards the identification of additional analogues suitable for *in vivo* studies and further mechanistic considerations are ongoing and will be reported in due course.
