# Supplementary material for: Potent bicyclic inhibitors of malarial cGMP-dependent protein kinase: approaches to combining improvements in cell potency, selectivity and structural novelty
Source: Bioorg Med Chem Lett. 2019 Oct 1;29(19):126610. doi: 10.1016/j.bmcl.2019.08.014 (PMC6857626; doi:10.1016/j.bmcl.2019.08.014)
Supplement: Supplementary data 1 [file mmc1.docx]

**Potent bicyclic inhibitors of malarial cGMP-dependent protein kinase: approaches to combining improvements in cell potency, selectivity and structural novelty**

Jonathan M. Large,^a,*^ Kristian Birchall,^a^ Nathalie S. Bouloc,^a^ Andy T. Merritt,^a^ Ela Smiljanic-Hurley,^a^ Denise J. Tsagris,^a^ Mary C. Wheldon,^a^ Keith H. Ansell,^a^ Peter J. Coombs,^a^ Catherine A. Kettleborough,^a^ David Whalley,^a^ Lindsay B. Stewart,^b^ Paul W. Bowyer,^b^ David A. Baker,^b^ Simon A. Osborne.^a^

^a^ Centre for Therapeutics Discovery, LifeArc, Accelerator Building, Open Innovation Campus, Stevenage, SG1 2FX, U.K.

^b^ Faculty of Infectious and Tropical Diseases, London School of Hygiene & Tropical Medicine, Keppel Street, London, WC1E 7HT, U.K.

**Supplementary information**

1. **General synthetic chemistry and analysis remarks**
2. **Synthesis and characterisation of key example compounds:**
   1. Compound **13**
   2. Compound **27**
3. **ADME assay procedures:**
   1. Measured LogD
   2. Mouse liver microsomal stability
   3. Kinetic solubility
4. **Docking procedure for compounds 2, 15, 16**
5. **Selectivity against human PKG orthologues**
6. **General synthetic chemistry and analysis remarks**

All commercial starting materials, reagents and solvents were used without further purification. Flash column chromatography was carried out using either an SP4 or Isolera-4 MPLC system (manufactured by Biotage) with pre-packed KP-silica gel cartridges (supplied by Biotage). ^1^H NMR spectra were obtained using a JEOL ECX400 spectrometer at room temperature; in all cases, NMR data were consistent with the proposed structures. Chemical shifts are given in parts-per-million referenced to residual undeuterated solvent peak, and conventional abbreviations used for multiplicity: e.g. s, singlet; d, doublet; t, triplet; q, quartet; dd, doublet of doublets; br, broad. Analytical mass spectra were recorded using a multi-mode ES+APCI mass spectrometer (6120 quadrupole LCMS manufactured by Agilent). Preparative high pressure liquid chromatography of final compounds was carried out using apparatus made by Agilent. The apparatus is constructed such that the chromatography (column: 19 x 100 mm (5 µm) Prep C-18 XBridge column at a flow rate of 40 ml/min) is monitored by a multi-wavelength UV detector (G1365B manufactured by Agilent) and a multi-mode ES+APCI mass spectrometer (G-1956A, manufactured by Agilent) connected in series, and if the appropriate criteria are met the sample is collected by an automated fraction collector (G1364B manufactured by Agilent). Collection can be triggered by any combination of UV or mass spectrometry, or can be based on time. Typical conditions for the separation process are as follows: the gradient is run over a 10-minute period (gradient at start: 10% methanol and 90% water, gradient at finish: 100% methanol and 0% water). For buffering, either 0.1% trifluoroacetic acid is added to the water (low pH buffer), or 0.1% ammonium hydroxide is added to the water (high pH buffer). It may be necessary or desirable to modify the conditions for a specific compound, for example by changing the solvent composition at the start or at the end, modifying the solvents or buffers, changing the run time, changing the flow rate and/or the chromatography column. For each compound purified, the collected fraction is analysed using four methods: either methanol or acetonitrile as the organic solvent, at either low pH (0.1% TFA, pH 1 or 0.1% formic acid, pH 3) or high pH (0.1% ammonium hydroxide, pH 10), and if necessary re-purified until ≥ 95% purity at 254 nm was achieved. This criterion was met for all compounds submitted for biological testing. Those obtained as TFA salts were desalted by passing through an Isolute “aminopropyl” NH_2_ cartridge (500 mg or 1 g size, eluting with three column volumes of a 2:1 CH_2_Cl_2_-MeOH solvent mixture), to give the free base before submitting for biological testing or ADME assays.

1. **Synthesis and characterisation of key example compounds**
2. Improved synthesis of compound **13**



(i) LiHMDS, ethyl cyclopropanecarboxylate, THF, -78 ^o^C; (ii) NBS, CH_2_Cl_2_, rt; (iii) 2-aminopyridine-4-methanol, EtOH, 4Å sieves, 100 ^o^C; (iv) *tert*-butyl 4-(4-aminophenyl)piperazine-1-carboxylate, TFA, s-BuOH, 100 ^o^C; (v) MsCl, pyridine, CH_2_Cl_2_, 0 ^o^C; (vi) Me_2_NH, THF, rt; (vii) TFA, CH_2_Cl_2_, 0 ^o^C - rt.

Preparation of compound **31**

To a solution of 2-chloro-4-methylpyrimidine **30** (5.0 g, 38.9 mmol) in THF (50 mL) was added LiHMDS (1M in THF, 117 mL, 117 mmol) dropwise at -78 °C, and the reaction stirred at the same temperature for 1 h. Then, a solution of ethyl cyclopropanecarboxylate (5.33 g, 46.7 mmol) in THF (10 mL) was added to the reaction mixture over a period of 10 min. The resulting reaction mixture was stirred at same temperature for 5 h. It was then quenched with saturated ammonium chloride solution and extracted with ethyl acetate (2 x 150 mL). The combined organic layer was washed with brine solution (100 mL) dried over Na_2_SO_4_ and concentrated *in vacuo*. Column chromatography (15% ethyl acetate in petroleum ether) to afford the product **31** (3.5 g, 46%) as a brown oil.

^1^H NMR (400 MHz, CDCl_3_) δ ppm 8.57 (d, J = 4.8 Hz, 1H), 7.25 (d, J = 5.2 Hz, 1H), 4.06 (s, 2H), 2.09 - 2.03 (m, 1H), 1.14 - 1.09 (m, 2H), 1.01 - 0.97 (m, 2H).

LC-MS (MeCN, pH 3): [M+H]^+^ = 197, 199, rt = 1.27 mins, purity = 89%.

Preparation of compound **32**

To a stirred solution of **31** (4.0 g, 20.3 mmol) in CH_2_Cl_2_ (60 mL) was added NBS (3.62 g, 20.3 mmol) portionwise at room temperature and the reaction stirred for 30 min. It was then quenched with saturated sodium bicarbonate solution and extracted with ethyl acetate (2 x 100 mL). The combined organic layer was washed with water (100 mL) dried over Na_2_SO_4_ and concentrated *in vacuo*. The obtained crude was purified by column chromatography (6% ethyl acetate in petroleum ether) to afford the intermediate bromoketone (4.0 g, 72%) as a brown oil.

^1^H NMR (400 MHz, CDCl_3_) δ ppm 8.69 (d, J = 5.2 Hz, 1H), 7.58 (d, J = 5.2 Hz, 1H), 5.54 (s, 1H), 2.31 - 2.25 (m, 1H), 1.26 - 1.13 (m, 4H).

LC-MS (MeCN, pH 3): [M+H]^+^ = 275, 277, 279, rt = 1.68 mins, purity = 84%.

To a stirred solution of the bromoketone (4.0 g, 14.5 mmol) in EtOH (100 mL) were added (2-aminopyridin-4-yl) methanol (1.8 g, 14.5 mmol) and molecular sieves (4Å, 8.0 g) at room temperature, and the reaction then stirred at 100 °C for 16 h. The precipitate was then filtered and washed with CH_2_Cl_2_ (100 mL), and the filtrate concentrated *in vacuo*. Trituration with ethanol then gave the product **32** (2.0 g, 46%) as a pale yellow solid.

^1^H NMR (400 MHz, DMSO-*d_6_*) δ ppm 9.41 (d, J = 7.2 Hz, 1H), 8.71 (d, J = 5.2 Hz, 1H), 7.96 (d, J = 5.6 Hz, 1H), 7.51 (s, 1H), 7.10 (dd, J = 7.2, 1.6 Hz, 1H), 5.50 (t, J = 5.6 Hz, 1H), 4.59 (d, J = 5.6 Hz, 2H), 2.38 - 2.35 (m, 1H), 1.11 – 1.09 (m, 4H).

LC-MS (MeCN, pH 3): [M+H]^+^ = 301, 303, rt = 1.16 mins, purity = 78%.

Preparation of compound **33**

To a stirred solution of **32** (500 mg, 1.66 mmol) in s-butanol (5 mL) were added *tert*-butyl 4-(4-aminophenyl)piperazine-1-carboxylate (498 mg, 1.82 mmol) and TFA (760 mg, 6.64 mmol) at room temperature. The resulting reaction mixture was stirred at 100 °C for 12 h. It was then diluted with 10% MeOH in CH_2_Cl_2_ (100 mL) and washed with saturated sodium bicarbonate solution. The organic layer was dried over Na_2_SO_4_ and concentrated *in vacuo*. Column chromatography (3% MeOH in CHCl_3_) to afford **33** (400 mg, 44%) as a pale yellow solid.

^1^H NMR (400 MHz, DMSO-*d_6_*) δ ppm 9.56 (s, 1H), 9.40 (s, 1H), 8.45 (d, J = 5.2 Hz, 1H), 7.58 (d, J = 9.2 Hz, 2H), 7.44 (s, 1H), 7.24 (d, J = 5.2, Hz, 1H), 6.95 – 6.88 (m, 3H), 5.45 (s, 1H), 4.58 (d, J = 3.2 Hz, 2H), 3.48 - 3.45 (m, 4H), 3.04 - 3.02 (m, 4H), 2.37 - 2.33 (m, 1H), 1.42 (s, 9H), 1.06 - 1.04 (m, 4H).

LC-MS (MeCN, pH 3): [M+H]^+^ = 542, rt = 1.62 mins, purity = > 95%.

Preparation of compound **34**

To a stirred solution of **33** (110 mg, 0.20 mmol) in pyridine (1 mL) was added methanesulfonyl chloride (47 mg, 0.40 mmol) at 0 °C, and stirred for 1 h. Then 2M dimethylamine (2M in THF, excess, 5 mL) was added to the reaction mixture, and stirring continued at room temperature for 1 h. The reaction was diluted with water and extracted with ethyl acetate. The organic layer was dried over Na_2_SO_4_ and concentrated *in vacuo*. The obtained crude was purified by column chromatography (3% MeOH in CHCl_3_) to afford **34** (70 mg, 60%) as a pale yellow solid.

^1^H NMR (400 MHz, DMSO-*d_6_*) δ ppm 9.51 (s, 1H), 9.37 (s, 1H), 8.46 (d, J = 5.2 Hz, 1H), 7.55 (d, J = 8.8 Hz, 2H), 7.42 (s, 1H), 7.24 (d, J = 5.2, Hz, 1H), 6.96 - 6.83 (m, 3H), 3.50 -3.46 (m, 6H), 3.04- (t, J = 5.2 Hz, 4H), 2.33 - 2.32 (m, 1H) 2.18 (s, 6H), 1.43 (s, 9H), 1.06 - 1.04 (m, 4H).

LC-MS (MeCN, pH 3): [M+H]^+^ = 569, rt = 1.59 mins, purity = 90%.

Preparation of compound **13**

To a solution of **34** (130 mg, 0.27 mmol) in CH_2_Cl_2_ (5 mL) was added TFA (0.5 mL) at 0 °C, and the reaction stirred at room temperature for 3 h. The reaction mixture was evaporated to dryness *in vacuo* and the residue basified with saturated sodium bicarbonate solution (5 mL) at 0 °C and extracted with CH_2_Cl_2_ (2 x 10 mL). The combined organic layer was dried over Na_2_SO_4_ and concentrated *in vacuo*. The crude compound was purified by preparative hplc to obtain the product **13** (30 mg, 28%) as an off-white solid.

^1^H NMR (400 MHz, DMSO-*d_6_*) δ ppm 9.52 (br s, 1H), 9.35 (s, 1H), 8.45 (d, J = 5.2 Hz, 1H), 7.54 (d, J = 8.8 Hz, 2H), 7.42 (d, J = 0.4 Hz, 1H), 7.22 (d, J = 5.6 Hz, 1H), 6.92 - 6.88 (m, 3H), 3.46 (s, 2H), 3.04 - 3.01 (m, 4H), 2.90 - 2.88 (m, 4H), 2.37-2.32 (m, 1H), 2.18 (s, 6H), 1.06 - 1.04 (m, 4H).

LC-MS (MeCN, pH 3): [M+H]^+^ = 469, rt = 1.01 mins, purity = > 95%.

1. Improved synthesis of compound **27**



(i) 2-amino-4-bromopyridine, EtOH, 4Å sieves, 80 ^o^C; (ii) BrettPhos palladacycle, RuPhos, Me_2_NCH_2_CH_2_NH_2_, Cs_2_CO_3_, ^t^BuOH, 70 ^o^C; (iii) H_2_O_2_, Na_2_WO_4_.2H_2_O, AcOH, MeOH, 0 ^o^C – rt, then CS_2_, CH_2_Cl_2_, MgSO_4_, rt; (iv) Me_2_NH, THF, 60 ^o^C.

Preparation of compound **35**

The bromoketone starting material **14** was prepared according to *Eur. J. Med. Chem.* **2007**, *42*, 1334.

To a flask containing the bromoketone **14** (11.0 g, 32.3 mmol) in dry ethanol (100 mL) under nitrogen was added 2-amino-4-bromopyridine (1.0 eq, 32.3 mmol, 5.6 g) and the reaction mixture stirred at 60 ^o^C for 18 h. After cooling, the formed precipitate was filtered off, washed with ethanol and dried under vacuum to give the product **35** (4.4 g, 33% yield) as an off-white solid.

^1^H NMR (400 MHz, DMSO-*d_6_*) δ ppm 9.25 - 9.23 (m, 1H), 8.47 (d, J = 5.0 Hz, 1H), 8.08 – 8.07 (m, 1H), 7.65 – 7.60 (m, 2H), 7.32 – 7.24 (m, 3H), 6.88 (d, J = 5.5. Hz, 1H), 2.55 (s, 3H).

LC-MS (MeCN, pH 10): [M+H]^+^ = 415, 417, rt = 2.01 mins, purity = > 95%.

Preparation of compound **36**

An oven-dried vial was charged with the bromide **35** (300 mg, 0.72 mmol, 1 eq), BrettPhos palladacycle (0.1 eq, 0.07 mmol, 58 mg), RuPhos (0.1 eq, 0.07 mmol, 34 mg) and cesium carbonate (3 eq, 2.26 mmol, 707 mg) and the vial capped and flushed with nitrogen. A solution of the amine N,N-dimethylethylenediamine (3 eq, 2.26 mmol, 0.24 mL) in ^t^BuOH (2 mL) was added, the solution degassed and heated at 70 ^o^C for 4 h. After cooling, concentration *in vacuo* directly onto silica and column chromatography (0 – 20% MeOH in EtOAc gradient) gave the product **36** (230 mg, 75%) as a yellow solid.

^1^H NMR (400 MHz, CDCl_3_) δ ppm 9.40 (d, J = 7.3 Hz, 1H), 8.17 (d, J = 5.5 Hz, 1H), 7.64 – 7.58 (m, 2H), 7.16 – 7.10 (m, 2H), 6.74 (d, J = 5.5. Hz, 1H), 6.55 (d, J = 2.8 Hz, 1H), 6.42 (dd, J = 7.6, 2.5 Hz, 1H), 4.91 (t, J = 4.6 Hz, 1H), 3.23 – 3.19 (m, 2H), 2.63 – 2.60 (m, 5H), 2.28 (s, 6H).

LC-MS (MeOH, pH 1): [M+H]^+^ = 424, rt = 1.13 mins, purity = > 90%.

Preparation of compound **27**

A solution of compound **36** (135 mg, 0.32 mmol) in MeOH (3 mL) at room temperature was treated with acetic acid (5 eq, 1.59 mmol, 0.09 mL), hydrogen peroxide (35% w/w in water, 4 eq, 1.30 mmol, 0.12 mL) and finally with sodium tungstate dihydrate (0.3 eq, 0.09 mmol, 31 mg) and the reaction stirred at room temperature for 1.5 h. It was then partitioned between dichloromethane and saturated sodium bicarbonate, the organic layer dried over magnesium sulfate and concentrated *in vacuo*. It was then re-dissolved in CH_2_Cl_2_ (3 mL) and treated with carbon disulfide (excess, 1 mL) and stirred at room temperature for 1 h. Then the magnesium sulfate was filtered off and the filtrate concentrated *in vacuo*, the residue treated with dimethylamine (2M in THF, 10 eq, 3.2 mmol, 1.6 mL) and stirred at 60 ^o^C for 3 h. Concentration *in vacuo* and purification of the residue by preparative hplc then gave the product **27** (55 mg, 42% yield) as an off-white solid.

^1^H NMR (400 MHz, DMSO-*d_6_*) δ ppm 9.38 (br s, 1H), 8.01 (d, J = 5.5 Hz, 1H), 7.62 – 7.57 (m, 2H), 7.29 – 7.23 (m, 2H), 7.19 (br s, 1H), 6.63 (dd, J = 7.6, 2.5 Hz, 1H), 6.43 (t, J = 5.0 Hz, 1H), 6.38 (d, J = 2.3 Hz, 1H), 6.20 (d, J = 5.0 Hz, 1H), 3.21 – 3.17 (m, 2H), 2.86 (br d, J = 2.8 Hz, 3H), 2.20 (s, 6H). Note that one signal for the second methylene group (2H) was not observed – we suspect this is obscured by the residual dmso solvent peak at 2.50 Hz.

LC-MS (MeOH, pH 10): [M+H]^+^ = 406, rt = 2.24 mins, purity = > 95%.

1. **ADME assay procedures**
   1. Measured logD

LogD measurements were carried out using the shake flask method. Compound was diluted from 10 mM DMSO stock solution into an eppendorf containing equal amounts of octanol and phosphate buffered saline (PBS) to give a final concentration of 100 μM. The tubes were shaken for 12 hours, centrifuged at 10000 rpm for 10 minutes and samples taken from the octanol and PBS layers. The samples from both layers were analysed in triplicate by LC-MS/MS (Agilent Technologies G6410 series, triple quadrupole with MM-ESI ion source) using optimised multiple reaction monitoring (MRM) scans and a standard column gradient on an Acquity UPLC BEH C8 1.7 μm column, running acetonitrile and water with 0.05% Acetic Acid as the mobile phase. The ratios of areas of the peaks were used to calculate the LogD in accordance with the equation:

LogD = Log_10_(Area-TL/Area BL)

- 1. mouse liver microsomal turnover

Microsomal turnover data was obtained using mouse liver microsomes (MLM) obtained from BD Biosciences. The compounds are pre-incubated at 37 °C for 5 minutes with the microsomes and the reaction was initiated by adding an equal volume of NADPH generating solution, also obtained from BD Biosciences. The final compound concentration in the incubation is 1 μM, and the microsomal protein concentration is 0.2 mg/mL. A sample is taken at t=0 and quenched with 2x volume of ice-cold methanol containing an internal standard reference compound (carbamazepine). The reaction is agitated at 37 °C for 30 minutes, when a further sample was taken and quenched in an identical fashion. The samples were centrifuged at 10000 rpm for 10 minutes and the supernatant taken for analysis in triplicate by LC-MS/MS (Agilent Technologies G6410 series, triple quadrupole with MM-ESI ion source) using optimised multiple reaction monitoring (MRM) scans and a standard column gradient on an Acquity UPLC BEH C8 1.7μm column, running acetonitrile and water with 0.05% acetic acid as the mobile phase. The % turnover is obtained by calculating the percentage difference of the peak areas, normalised to the internal standard, at t=0 and t=30. Verapamil is used as the standard compound for this assay.

- 1. Kinetic solubility

The kinetic solubility was measured by diluting a small amount of 10 mM DMSO stock into PBS pH7.4 in a filtration plate at a target concentration of 200 μM giving a final solution composition of 98:2 PBS:DMSO. Each compound was run in triplicate on the same plate with two standard compounds, verapamil and ketoconazole, included per plate. The filtration plate was shaken at 500 rpm for 90 minutes and then filtered under vacuum. The filtrate was sampled and diluted with a DMSO:PBS mixture in a flat bottomed UV plate to give a solution with a composition of PBS:DMSO 80:20. A dilution series for each compound was then created in flat bottomed UV plates in PBS:DMSO 80:20 with concentrations 200 μM, 100 μM, 50 μM, 25 μM, 12.5 μM & 6.75 μM. The UV absorbance for these solutions was read across 230-400 nm at 1 nm intervals using a TECAN Safire II plate reader and a suitable UV wavelength chosen around the UV maximum of each compound. This was used to calculate the concentration in the filtrate for each compound, and hence amount remaining in solution after 90 minutes which is reported as the kinetic solubility.

1. **Docking procedure for compounds 2, 15, 16**

Docking studies were carried out using the Schrodinger suite. Compounds were prepared using the LigPrep utility to generate a low energy conformation, with ionisation state at pH 7.4 assigned using Epik. The protein (PDB:5DYK) was prepared using the Protein Preparation Wizard with default parameters, including assignment of bond orders, tautomer and ionisation states, addition of hydrogen atoms, removal of water molecules, optimisation of H-bonding networks and restrained minimisation using the OPLS2005 forcefield. Residues A823 and E824 immediately preceding a stretch of amino acids not visible in the model were removed because of their poor fit to the electron density, to avoid their unreliable positioning confounding the docking results. The grid for docking was generated using a 12-angstrom inner box and 32-angstrom outer box centred on the ATP binding site, with H-bond constraints to the peptide backbone of hinge residue V621. Docking was carried out using GlideSP (rigid receptor, fully flexible ligand) with default parameters, including strain correction terms and outputting up to 5 poses. The lowest energy poses were consistent between ligands, building confidence in the binding mode hypothesis.

1. **Selectivity against human PKG orthologues**

Compounds **2** and **27** were tested externally in a Millipore Kinase Profiler assay (see www.merckmillipore.com for further details) against the two human PKG orthologues, denoted as PKG1α(h) and PKG1β(h). pIC_50_ values for the two compounds were determined as shown below:

| **Compound** | **Kinase** | **pIC_50_** |
| --- | --- | --- |
| **2** | PKG1α(h) | < 6 |
|  | PKG1β(h) | < 6 |
| **27** | PKG1α(h) | < 6 |
|  | PKG1β(h) | < 6 |
